# A novel of new class II bacteriocin from *Bacillus velezensis* HN-Q-8 and its antibacterial activity on *Streptomyces scabies*

**DOI:** 10.3389/fmicb.2022.943232

**Published:** 2022-07-29

**Authors:** Jing Zhao, Zhijun Zhou, Xuefei Bai, Dai Zhang, Likui Zhang, Jinhui Wang, Beibei Wu, Jiehua Zhu, Zhihui Yang

**Affiliations:** ^1^College of Plant Protection, Hebei Agricultural University, Baoding, China; ^2^Technological Innovation Center for Biological Control of Crop Diseases and Insect Pests of Hebei Province, Baoding, China; ^3^Experimental Training Center of Hebei Agricultural University, Baoding, China; ^4^College of Environmental Science, Yangzhou University, Yangzhou, China

**Keywords:** potato, potato common scab, *Streptomyces scabies*, Lcn972, *Bacillus*

## Abstract

Potato common scab is a main soil-borne disease of potato that can significantly reduce its quality. At present, it is still a challenge to control potato common scab in the field. To address this problem, the 972 family lactococcin (Lcn972) was screened from *Bacillus velezensis* HN-Q-8 in this study, and an *Escherichia coli* overexpression system was used to obtain Lcn972, which showed a significant inhibitory effect on *Streptomyces scabies*, with a minimum inhibitory concentration of 10.58 μg/mL. The stability test showed that Lcn972 is stable against UV radiation and high temperature. In addition, long-term storage at room temperature and 4°C had limited effects on its activity level. The antibacterial activity of Lcn972 was enhanced by Cu^2+^ and Ca^2+^, but decreased by protease K. The protein was completely inactivated by Fe^2+^. Cell membrane staining showed that Lcn972 damaged the cell membrane integrity of *S. scabies*. Scanning electron microscope (SEM) and transmission electron microscope (TEM) observations revealed that the hyphae of *S. scabies* treated with Lcn972 were deformed and adhered, the cell membrane was incomplete, the cytoplasm distribution was uneven, and the cell appeared hollow inside, which led to the death of *S. scabies*. In conclusion, we used bacteriocin for controlling potato common scab for the first time in this study, and it provides theoretical support for the further application of bacteriocin in the control of plant diseases.

## Introduction

As a main soil-borne disease of potato, common scab has caused a serious deterioration in the quality of potato in recent years. Potato common scab can be caused by a variety of pathogenic *Streptomyces*, and *S. scabies*, *S. acidiscabies*, *S. turgidiscabies*, and *S. ipomoeae* are often found in the field (Shi et al., 2019). Among them, *S. scabies* is the most common pathogenic bacteria and can cause the most typical symptoms (Lin et al., 2018). At present, the effective control of common scab of potato is a huge challenge because conventional methods only reduce the abundance of pathogenic bacteria (Wanner, 2006; St-Onge et al., 2008; Dees and Wanner, 2012; Zhang et al., 2016).

In recent years, bacteria such as *Pseudomonas* spp. and *Bacillus* spp. have been studied and used as bio-control agents for plant diseases (Chowdhury et al., 2015; Balthazar et al., 2022). Cui et al. (2020) showed that the control efficiency of *Bacillus velezensis* 8-4 against potato common scab reached 51.83 ± 8.53% in field experiments. *Bacillus*-based biological control become a focus in the prevention and control of potato common scab because of its high activity against the pathogenic bacteria, environmental friendliness, and broad antibacterial spectrum (Meng et al., 2016; Lin et al., 2018).

It is generally believed that the bio-control mechanism of *Bacillus* includes antibiosis, competition for nutrients and niches, for which antibiosis is an important way to exert its bio-control activity (Dimkić et al., 2022). As a bio-control weapon, *Bacillus* can secrete antibacterial substances, such as lipopeptides, phenols, proteases, and short peptides. In total, 4.5–15.4% of the *Bacillus* genes are involved in producing anti-bacterial compounds (Chen et al., 2009; Shafi et al., 2017). Among these compounds, bacteriocins have strong antibacterial activites. They are a kind of active polypeptide or precursor polypeptide, synthesized through the ribosomal pathway during bacterial metabolism, and it has a strong inhibitory effect on gram-positive bacteria (Yi et al., 2016; Garcia-Gutierrez et al., 2020). As a type of natural antibacterial substances, bacteriocins are considered promising alternatives to antibiotics and have broad application prospects in food, medicine, and agriculture because they are relatively safe to humans and animals, and they do not easily confer resistance in their targets (Cotter et al., 2013; Kamarajan et al., 2015; Kumariya et al., 2019).

Almost all bacteria can synthesize bacteriocins, thereby establishing a substantial basis for their development and application (Rooney et al., 2020). The molecular weight, activity and properties of different bacteriocins vary widely. They can be divided into lantibiotics and non-lantibiotics depending on whether the sequence contains the unusual amino acids lanthionine and methyllanthionine (Rizzello et al., 2014). In addition, bacteriocins can be divided into four classes based on differences in structure and properties (Ennahar et al., 2000). Class I bacteriocins are post-translationally modified small linear peptides, usually less than 5 KD, with membrane activity. Class II bacteriocins are thermostable peptides with membrane activity and hydrophobic activity that are usually less than 10 KD. Class III bacteriocins are heat-sensitive macromolecule proteins that are usually more than 30 KD. Class IV bacteriocins are complex macromolecular bacteriocins, containing carbohydrate or lipid groups in addition to protein (Klaenhammer, 1993; Zou et al., 2018). Among them, Class I and II bacteriocins have been studied as food preservatives owing to their high activity and specificity to target strains (Delves-Broughton et al., 1996; Wiedemann et al., 2001).

The antibacterial mechanisms of bacteriocins such as nisin, sakacin, and pedioncin PA-1 from *Escherichia coli* and *Lactococcus lactis* have been revealed in depth. However, the antibacterial mechanisms of bacteriocins secreted by *Bacillus* still need further investigation (Todorov et al., 2011). The bacteriocins secreted by *Bacillus* are mainly Class I and II (Barbosa et al., 2015), such as ericin S, BacBS2 and BLIS by *B. velezensis* and amylocyclicin, plantazolicin, and amysin by *Bacillus amyloliquefaciens* (Scholz et al., 2011, 2014; Kaewklom et al., 2013; Palazzini et al., 2016; Butkhot et al., 2019; Perumal et al., 2019; Vaca et al., 2019), which can cause cell membrane perforation, and increase the cell membrane permeability and leakage of cytoplasmic components, leading to bacterial death (Szabo and Cahill, 2002; Pandey et al., 2013; Kamarajan et al., 2015; Kumariya et al., 2015; Ekblad et al., 2016; O Connor et al., 2018). With the development of sequencing technology, many bacteriocins have been identified and studied, and the study of their antibacterial mechanisms is crucial for the further applications.

The antibacterial activities of bacteriocins have been broad applied in food preservation and feed additives (Mathur et al., 2018; Wang et al., 2021), whereas, the application of bacteriocins in the control of pathogenic bacteria in agriculture has only been rarely reported. Studying and utilizing bacteriocins in the control of plant diseases is of great significance to their development and application, as well as the bio-control of plant diseases. *Bacillus velezensis* HN-Q-8 was isolated from soil, and it has a strong antibacterial activity against *S. scabies* (Supplementary Figure 1). It can secrete various antagonistic substances, such as lipopeptides and proteins, and it showed strong bacteriostatic effects in both laboratory and field tests. In our study, we screened bacteriocin from *B. velezensis* HN-Q-8 for the treatment of potato common scab, and the results provide a material basis and theoretical support for the control of potato common scab.

## Materials and methods

### Screening of the bacteriocin

Gene clusters in *B. velezensis* HN-Q-8 (CP045711.1) were predicted by an online website tool antiSMASH^1^ to screen for bacteriocin-coding gene. The bacteriocin sequence was searched in Uniport^2^ and NCBI.^3^ Then phylogenetic tree of the bacteriocin sequence and its homologous gene sequences was constructed to classify the bacteriocin. The protein sequence of bacteriocin was compared with homologous bacteriocins secreted by other *Bacillus*.

### Bacterial strains and culture conditions

The bacterial strains and the culture conditions used in this study are provided in Table 1, and the information of the bacterial strains are shown in Supplementary Table 1. All the bacterial strains were stored at −80°C in LB or water with 30% glycerol solution until use.

**TABLE 1:** Bacterial strains and culture conditions.

Indicator strains	Culture medium	Incubation temperature
*Bacillus pumilus* G15	LB	37°C
*Bacillus subtilis* 3610	LB	37°C
*Bacillus amyloliquefaciens* Z17-2	LB	37°C
*Bacillus atrophaeus* YN-29	LB	37°C
*Bacillus mycoides* Y-7	LB	37°C
*Bacillus licheniformis* J-5	LB	37°C
*Bacillus thuringiensis* 2.19	LB	37°C
*Bacillus cereus* 2.15	LB	37°C
*Bacillus velezensis* FX	LB	37°C
*Bacillus mojavensis* C28	LB	37°C
*Streptomyces turgidiscabies* HY9	Gauze’s synthetic broth medium/OMA	28°C
*Streptomyces stelliscabiei* FN1	Gauze’s synthetic broth medium/OMA	28°C
*Streptomyces scabies* HP4	Gauze’s synthetic broth medium/OMA	28°C
*Streptomyces europaeiscabiei* MY	Gauze’s synthetic broth medium/OMA	28°C
*Pectobacterium brasiliense* 412	LB	28°C
*Pectobacterium atrosepticum* B412	LB	28°C

### Production of bacteriocin

An *E. coli* expression system was used to obtain the recombinant bacteriocin. The bacteriocin-encoding gene was amplified from the genomic DNA of *B. velezensis* HN-Q-8 by PCR using the following primers pair: 5′-GGGAATTCCATATGTTGAAAAAGAAGGTTCTTGCT-3′/5′ -CCGCTCGAGTTTTGTATCCCAAAAAGCCTT-3′. The amplified fragment and the pET-30a vector were double digested using NdeI and XhoI endonucleases, and then ligated with pET-30a using T4 ligase. The bacteriocin was expressed from pET-30a in *E. coli* BL21 and then collected from the cell lysate supernatant (crude bacteriocin). The antibacterial activity of the cell lysate supernatant against *S. scabies* HP4 was tested using the agar well diffusion method (Parente et al., 1995). In total, 100 μL 1.0 × 10^7^ CFU/mL of *S. scabies* HP4 spores were spread on oatmeal agar (OMA) solid medium. Then 6-mm hole was punched into the medium, and 70 μL cell lysate supernatant was added to the hole (Yi et al., 2018).

### Purification and identification

The cell lysate supernatant was heated independently at 30, 40, 50, 60, 70, 80, 90, 100, and 121°C for 30 min and then centrifuged at 18,000 × *g* for 30 min. Then, the supernatant was filtered using a 0.22-μm filter (Jin Teng, China) and loaded on a Ni-NTA column (Solarbio, Beijing, China) (Jiang et al., 2021). Five column volumes of Buffer B (500 mM NaCl, 20 mM Tris–HCl, 10% glycerol, 80 mM imidazole, pH 8.0) were used to wash the column, and the bound bacteriocin was eluted using five column volumes of Buffer C (500 mM NaCl, 20 mM Tris–HCl, 10% glycerol, 500 mM imidazole, pH 8.0). The eluted protein was verified using 15% SDS-PAGE. Finally, eluted protein fractions were dialyzed using a dialysis bag with MW 3,500 (Solarbio, Beijing, China) in Buffer A (500 mM NaCl, 20 mM Tris–HCl, 10% glycerol, pH 8.0). Purified bacteriocin was verified by LC-MS/MS analysis (Sangon Biotech, Shanghai, China).

### Antibacterial spectrum of the bacteriocin

The antibacterial spectrum of the bacteriocin was determined using *Bacillus* spp., *Streptomyces* spp., and *Pectobacterium* spp., which are listed in Table 1. In total, 100 μL 1.0 × 10^9^ CFU/mL of *Bacillus* spp. and *Pectobacterium* spp. were spread on LB agar plates, independently. And 100 μL 1.0 × 10^7^ CFU/mL of *Streptomyces* spp. spores were spread on OMA agar plates. The bacteriocin activity was tested using the agar well diffusion method, in which 70 μL of bacteriocin was added into 6-mm hole in the solid medium (Zhang et al., 2018).

### Minimum inhibitory concentration and optimal inhibitory concentration of the bacteriocin

The bacteriocin was diluted independently 10-fold and 2-fold with Buffer A, and then, the minimum inhibitory concentration (MIC) and optimal inhibitory concentration were determined using the agar well diffusion method. In total, 10^6^ CFU of *S. scabies* HP4 spores were spread on OMA solid medium, and 6-mm holes were punched into the agar. The holes were filled with 70 μL Lcn972. After the plates were incubated at 28°C for 7 days, the diameters of the inhibition zones were measured. MIC was defined as the minimum concentration of bacteriocin that formed a zone of inhibition on OMA solid medium (Chen et al., 2017). The optimal inhibitory concentration was defined as the concentration at which the zone of inhibition no longer increased significantly with increasing concentrations of bacteriocin on OMA agar plates.

### Stability of the bacteriocin

To evaluate the stability of bacteriocin, we tested the effects of salt concentration, pH, UV, proteases, and metal ions on the activity of bacteriocin, as follows:

To determine the effects of salinity, 7, 11, 15, 19, 23, 27, and 31% (m/V) NaCl solutions were prepared and mixed with bacteriocin (3% NaCl in Buffer A) in equal volumes to prepare final concentrations of 5, 7, 9, 11, 13, 15, and 17% NaCl solution environments, respectively. Then the antibacterial activities of bacteriocins in different NaCl solutions against *S. scabies* HP4 were determined to clarify the effect of NaCl concentration on the activity of bacteriocin. Bacteriocin added sterile water was used as positive control (CK), and the same concentrations of NaCl were used as negative controls.

The pH of bacteriocin was adjusted to 3, 4, 5, 6, 7, 8, 9, 10, and 11 using 5 mol/L HCl and NaOH solutions. The samples were then incubated at 37°C for 30 min, and then, the pH was adjusted back to eight. The stability of bacteriocin in acid and base conditions was then determined.

To reveal the sensitivity of bacteriocin to various proteases, trypsin, pepsin, proteinase K, alkaline protease, papain, and chymotrypsin were selected. The proteases were each mixed to a final concentration of 2 mg/mL with bacteriocin. The samples were incubated at 37°C for 30 min and then at 100°C for 10 min to inactivate the protease. Bacteriocin without protease was used as a control.

In total, 1 moL/L FeSO_4_, CaCl_2_, MgSO_4_, ZnSO_4_, MnSO_4_, CuSO_4_, and KCl were prepared and independently added to bacteriocin (producing a final concentration of 10 mmol/L) to reveal the sensitivity of bacteriocin to metal ions.

Additionally, to evaluate the sensitivity of bacteriocin to storage, the bacteriocin was stored at 4°C and room temperature for 16 days. The untreated bacteriocin was used as a control.

After the above treatments, the residual activities of all the samples were determined using the agar well diffusion method with *S. scabies* HP4 as the indicator strain.

### Cell membrane integrity

*Streptomyces scabies* HP4 was cultured in Gauze’s synthetic broth medium for 5 days at 28°C, centrifuged at 18,000 × *g* for 10 min to collect mycelium. The collected mycelium was suspended in 1 × phosphate buffer saline (PBS), added bacteriocin with a final concentration of 2 × MIC, incubated at 28°C for 3 h, then the mycelium was washed and resuspended in 1 × PBS. Then DiSC3-(5) was added into the mycelium to a final concentration of 30 μmol/L and incubated for 5 min until maximum absorption. The fluorescence intensity was observed under the excitation wavelength of 651 nm and the emission wavelength of 675 nm using a laser confocal microscope (Olympus, Tokyo, Japan). The mycelium added Buffer A as negative control, and kanamycin and Triton X-100 as positive controls.

### Scanning electron microscope and transmission electron microscope

Scanning electron microscope (SEM) and transmission electron microscope (TEM) were performed to monitor the morphological and structural changes of *S. scabies* HP4 cells after being treated with 9 × MIC of bacteriocin on OMA solid medium. For the SEM analysis, *S. scabies* HP4 was sampled at the edge of the inhibition zone, in the inhibition zone and in the non-inhibition zone on OMA solid medium. Each sample was fixed in 2.5% glutaraldehyde for 4 h, then dehydrated in ethanol and tert-butanol, respectively, and finally, the samples were freeze-dried and sputter-coated with gold. Photomicrographs of cells were observed using a ThermoFisher Prisma E Scanning Electron Microscope (ThermoFisher, MA, United States). For the TEM, the samples were fixed in 2.5% glutaraldehyde and 1% osmic acid and then dehydrated in ethanol. Afterward, the samples were permeated in resin and cut into sections. They were stained with 2% uranyl acetate and lead citrate. The sections were observed using a Hitachi H-7650 Transmission Electron Microscope (Hitachi, Tokyo, Japan).

### Effect of *Streptomyces scabies* HP4 on the expression of the bacteriocin

In total, 15 mL supernatant of *S. scabies* HP4 (cultured in Gauze’s synthetic broth medium) was added to 100 mL culture solution of *B. velezensis* HN-Q-8 and incubated at 37°C for 24 h. The relative expression of the bacteriocin was determined at 6 and 24 h using the following primer pair: 5′-CAGTCAGTCGAACGATGTCAA-3′/5′-TCGAAGCCATGATACCAAGT-3′.

### Statistical analysis

The data from experiments were calculated by measuring three independent replicates using IBM SPSS Statistics 19.0. A one-way analysis of variance was used to analyze the significance levels of the differences between the means. In all the analysis, a *P*-value of 0.05 was considered significant.

## Results

### Obtaining of the bacteriocin

#### Prokaryotic expression of the bacteriocin

**FIGURE 1:**
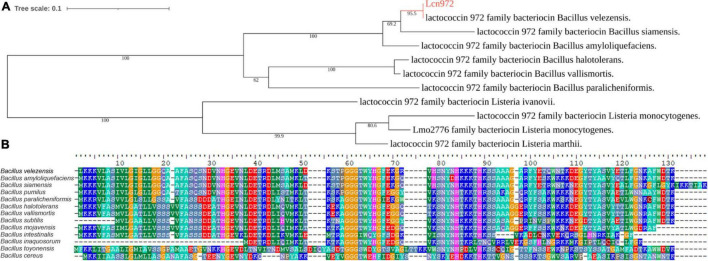
Lcn972 secreted by *Bacillus velezensis* HN-Q-8. **(A)** Phylogenetic analysis of Lcn972; **(B)** comparison of the amino acid sequence of Lcn972 from *B. velezensis* HN-Q-8 with these of Lcn972 from other *Bacillus* spp.

By analyzing the gene clusters of *B. velezensis* HN-Q-8 (Supplementary Figure 2A), a RiPP-like encoding sequence in Region 6 was found (Supplementary Figures 2B,C). The details of RiPP-like encoding sequence were analyzed (Supplementary Figure 2D), and a sequence homolog of 972 family Lactococcin (Lcn972) was identified (LKKKVLASIVLGIGLLGGQAAFASQSNDVNHGEVNLDESR DLMSAMKLDKSTPGGGTWYHGFEKGRVHSNYNHKKKT HKSSAAAGARFYETQWNTKDEGYTYASVYETLFGNKAFW DTK) (Figure 1A). The sequence of Lcn972 in *B. velezensis* HN-Q-8 was obviously mutated compared with the bacteriocins’ sequences of 972 family members secreted by other *Bacillus* (Figure 1B).

**FIGURE 2:**
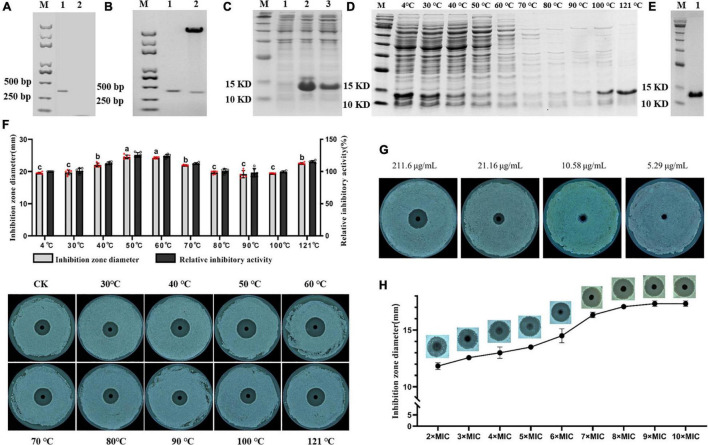
Obtain of Lcn972 and its antibacterial activity. **(A)** Obtaining of Lcn972. Lane 1, Lcn972; Lane 2, control; **(B)** double-digestion verification. Lane 1, Lcn972; Lane2, double digestion of pET-30a-Lcn972; **(C)** expression of Lcn972. Lane 1, before expression of Lcn972; Lane 2, after expression of Lcn972; Lane 3, total protein containing Lcn972; **(D)** total protein containing Lcn972 purified by heating; **(E)** Lcn972 purified by nickel column; **(F)** antibacterial activity of total protein containing Lcn972 on *Streptomyces scabies* HP4 after heating; **(G)** minimum inhibitory concentration (MIC) of Lcn972 against *S. scabies* HP4, **(H)** optimal inhibitory concentration of Lcn972 against *S. scabies* HP4. Letters on the graph denote statistically significant differences (ANOVA, *P* < 0.05).

Lcn972 was cloned, double-enzyme digested (Figure 2A) and ligated into pET-30a to construct pET-30a-Lcn972. pET-30a-Lcn972 was transformed into *E. coli* DH5α competent cells, and then, the plasmid was extracted and verified by double-enzyme digestion (Figure 2B). Sequencing was performed to detect whether the sequence was mutated, and then, pET-30a-Lcn972 was transformed into *E. coli* DE3 BL21 competent cells. The overexpressed protein band was observed on SDS-PAGE after inducible expression (Figure 2C).

#### Determination of the activity of Lcn972 against *Streptomyces scabies*

Total protein containing Lcn972 (crude bacteriocin Lcn972) of the *E. coli* BL21 was extracted, and the antibacterial activity against *S. scabies* HP4 was determined (Supplementary Figure 3). The total protein containing Lcn972 had a strong bacteriostatic activity on *S. scabies* HP4, whereas the protein without Lcn972 showed no bacteriostatic effect on *S. scabies* HP4.

#### Purification of Lcn972

The purification was first carried out by heating, as shown in Figure 2D. As the temperature increased from 30 to 90°C, the amount of the Lcn972 decreased, while the content of the Lcn972 increased when temperatures greater than 100°C. After 121°C heating for 30 min, Lcn972 alone could be seen on the SDS-PAGE. The antibacterial activity of Lcn972 treated at different temperatures against *S. scabies* HP4 increased at temperatures less than 50°C, and then decreased and finally increased again. The activity of Lcn972 was fully retained after heating to 121°C (Figure 2F). Then, Lcn972 was purified using nickel column affinity chromatography, as shown in Figure 2E, the purity and concentration of Lcn972 were great enough to determine the antibacterial activity.

#### Antibacterial spectrum of Lcn972

To reveal the effect of Lcn972 on soil bio-control bacteria and pathogenic bacteria, the antibacterial activities against *Bacillus* spp., *Streptomyces* spp., and *Pectobacterium* spp. were determined. As shown in Table 2 and Supplementary Figure 4, Lcn972 showed significantly antibacterial activities against gram-positive *Bacillus* spp. and *Streptomyces* spp., but no activity against *Pectobacterium* spp.

**TABLE 2:** Antibacterial activity of Lcn972 against bacteria.

Indicator strains	Inhibition zone diameter	Inhibitory effect
*Bacillus pumilus* G15	9.08 ± 0.14	+
*Bacillus subtilis* 3610	15.08 ± 1.42	++
*Bacillus amyloliquefaciens* Z17-2	11.67 ± 1.26	++
*Bacillus atrophaeus* YN-29	12.25 ± 0.35	++
*Bacillus mycoides* Y-7	8.58 ± 0.80	+
*Bacillus licheniformis* J-5	12.25 ± 0.43	++
*Bacillus thuringiensis* 2.19	8.17 ± 0.29	+
*Bacillus cereus* 2.15	4.67 ± 0.14	+
*Bacillus velezensis* FX	14.50 ± 0.90	++
*Bacillus mojavensis* C28	15.00 ± 0.50	++
*Streptomyces turgidiscabies* HY9	18.92 ± 0.80	++
*Streptomyces stelliscabiei* FN1	19.92 ± 0.95	++
*Streptomyces europaeiscabiei* MY	22.00 ± 1.00	+++
*Pectobacterium brasiliense*	−	−
*Pectobacterium atrosepticum*	−	−

Values are presented as the means ± standard deviations (n = 3). “+” indicates antibacterial activity and “−” indicates no antibacterial activity.

### Minimum inhibitory concentration and optimal inhibitory concentration of Lcn972

The MIC and optimal inhibitory concentration of Lcn972 against *S. scabies* HP4 were determined by serial dilution. The MIC was 10.58 μg/mL (Figure 2G), and the optimal inhibitory concentration was 95.22 μg/mL (9 × MIC) (Figure 2H).

### Sensitivity of Lcn972

#### Effects of NaCl concentration on the antibacterial activity of Lcn972

**FIGURE 3:**
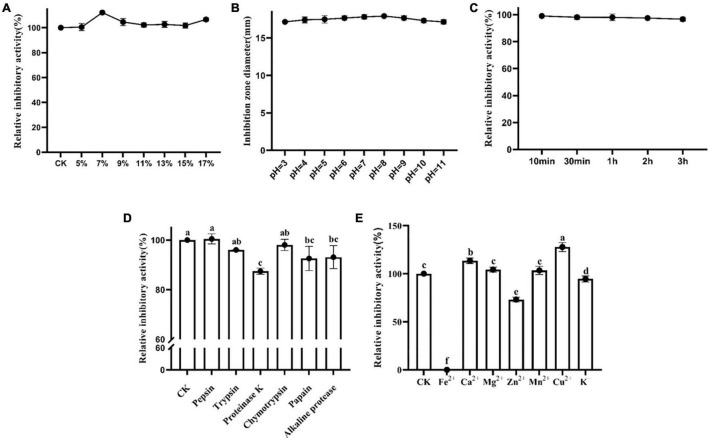
Sensitivity of Lcn972. **(A)** Effect of NaCl concentration on the antibacterial activity of Lcn972; **(B)** effect of pH on the antibacterial activity of Lcn972; **(C)** sensitivity of Lcn972 to UV; **(D)** sensitivity of Lcn972 to proteases; **(E)** sensitivity of Lcn972 to metal ions. Letters on the graph denote statistically significant differences (ANOVA, *P* < 0.05).

To reveal the effects of salt concentration on the activity of Lcn972, Lcn972 was treated with different concentrations of NaCl. The results are as shown in Figure 3A. As the NaCl concentration increased, the antibacterial activity of Lcn972 against *S. scabies* HP4 increased first, then decreased and finally increased again. Lcn972 showed its strongest antibacterial activity at a 7% NaCl concentration, with an increase of 12.50% compared with the control. This indicated that 7% NaCl was the optimal concentration for Lcn972 to exert its activity. Although the activity of Lcn972 increased when the NaCl concentration was 17%, there was an inhibitory effect of the high NaCl concentration on *S. scabies* HP4 (Supplementary Figure 5).

#### Effect of pH on the antibacterial activity of Lcn972

The antibacterial activity of Lcn972 on *S. scabies* HP4 after being treated at different pH levels was determined. The results are shown in Figure 3B. Lcn972 was stable in both strong acid and strong basic conditions, and the antibacterial activity of Lcn972 did not change significantly.

#### Sensitivity of Lcn972 to UV light

Lcn972 still maintained a strong antibacterial activity after UV irradiation (Figure 3C). As the UV irradiation time increased, the antibacterial activity of Lcn972 against *S. scabies* HP4 decreased slightly. Compared with the control, the antibacterial activity still maintained a 96.71% level after UV irradiation for 3 h, which indicated that Lcn972 is not a UV-sensitive bacteriocin.

#### Sensitivity of Lcn972 to proteases

To clarify the sensitivity of Lcn972 to proteases, Lcn972 was treated with six different proteases independently, and then, the antibacterial activity against *S. scabies* HP4 was determined. As shown in Figure 3D, the activity of Lcn972 treated with proteinase K was significantly reduced, decreasing by 12.56% compared with the control. In addition, the antibacterial activity decreased by 7.43 and 6.91% after treatment with papain and alkaline protease, respectively. The other proteases had limited effects on the antibacterial activity of Lcn972.

#### Sensitivity of Lcn972 to metal ions

Metal ions have a significant effect on protein activity (Figure 3E). In our study, Lcn972 was completely inactivated by Fe^2+^. In addition, Zn^2+^ and K^+^ significantly reduced the bacteriostatic activity of Lcn972, to 73.86 and 94.60%, respectively. However, the antibacterial activity of Lcn972 was significantly enhanced by Ca^2+^ and Cu^2+^, and the relative antibacterial activities increased to 113.54 and 127.79%, respectively. Other metal ions had no significant effects on the activity of Lcn972.

#### Effect of storage on the activity of Lcn972

**FIGURE 4:**
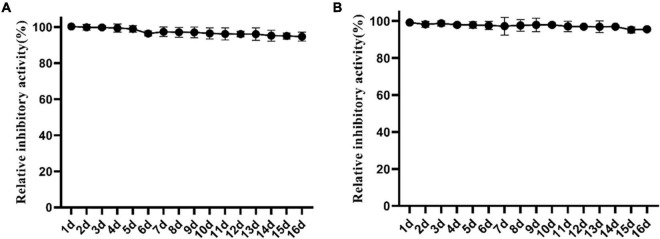
Effect of storage at different temperatures on the activity of Lcn972. **(A)** 4°C; **(B)** room temperature.

To clarify the effect of storage on the antibacterial activity of Lcn972 against *S. scabies* HP4, Lcn972 was stored at room temperature and 4°C and its activity was examined. As shown in Figure 4, the antibacterial activity against *S. scabies* HP4 was not notably reduced after storage at room temperature and 4°C for 16 days. Compared with the control, the antibacterial activity of Lcn972 against *S. scabies* HP4 storage at room temperature and 4°C decreased to 95.55 and 94.70%, respectively. This indicated that Lcn972 was stable after purification and can handle long-term storage.

### Antibacterial mechanism of Lcn972

**FIGURE 5:**
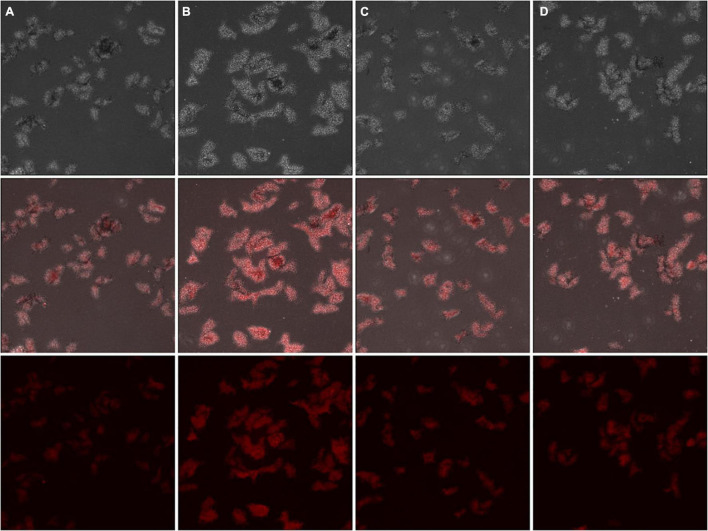
Fluorescent staining of cell membranes of *Streptomyces scabies* HP4 treated with Lcn972. The mycelia were treated with **(A)** Buffer A (negative control); **(B)** 60 ng/L kanamycin (positive control); **(C)** 0.5% TritonX-100 (positive control); **(D)** 2 × MIC Lcn972. Each column represents one sample.

To clarify the anti-bacterial mechanism of Lcn972 on *S. scabies*, the effect of Lcn972 on the integrity of the cell membrane was detected. The cell membrane of *S. scabies* HP4 was stained with DiSC3-(5), and the fluorescence of *S. scabies* were enhanced compared with the control after treatment with Lcn972 (Figure 5). This indicated that Lcn972 reduced the integrity of the cell membrane of *S. scabies* HP4.

**FIGURE 6:**
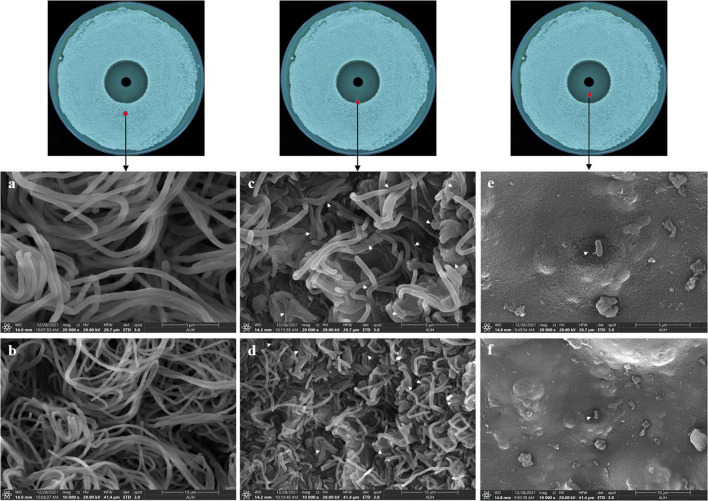
Scanning electron micrographs of *S. scabies* treated with Lcn972. (a,b): non-inhibition zone; (c,d): edge of the inhibition zone; (e,f): inhibition zone.

**FIGURE 7:**
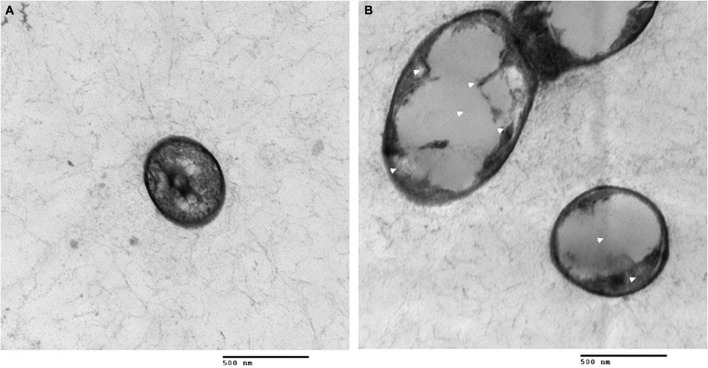
Transmission electron micrographs of *S. scabies* treated with Lcn972. **(A)** without Lcn972; **(B)** with Lcn972.

To further reveal the effects of Lcn972 on the cell structure of *S. scabies*, SEM and TEM were used to observed *S. scabies* HP4 treated with Lcn972. As shown in Figure 6, the hyphae of *S. scabies* HP4 were deformed, bent and shrunken. Additionally, the hyphae were adhered and seriously sunken. The growth of *S. scabies* was inhibited after being treated with Lcn972. The hyphae of the control were straight, with less curl and depression. The TEM revealed that the cell structure of *S. scabies* not treated with Lcn972 was compact, the cytoplasm was evenly distributed, and the membrane structure was complete (Figure 7A). Significant changes were found in the interiors of cells treated with Lcn972. The cytoplasmic distribution of *S. scabies* HP4 was uneven, the cell membrane was incomplete, and the cell structure was severely damaged (Figure 7B). This indicated that Lcn972 damaged both the cell interior and cell membrane of *S. scabies* HP4 cells.

### Effect of *Streptomyces scabies* on the expression of Lcn972

**FIGURE 8:**
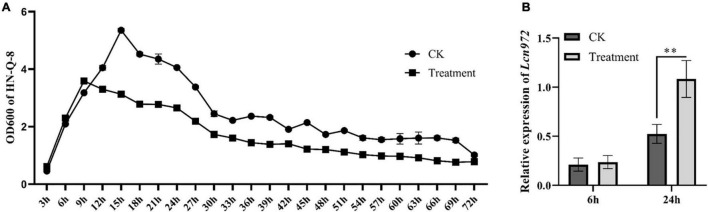
Effect of *Streptomyces scabies* on the expression of Lcn972. **(A)** Growth curve of *Bacillus velezensis* HN-Q-8; **(B)** relative expression of Lcn972. CK: *B. velezensis* HN-Q-8 added to Gauze’s synthetic broth medium; Treatment: *B. velezensis* HN-Q-8 added to cell-free culture medium of *S. scabies* HP4. **P < 0.01, one-way ANOVA, followed by Dunnet post-test, compared to control.

The supernatant of *S. scabies* HP4 was added to LB broth to culture *B. velezensis* HN-Q-8. Then, the relative expression level of Lcn972 from *B. velezensis* HN-Q-8 was determined in the logarithmic growth phase (6 h) and stationary phase (24 h). As shown in Figure 8, the expression of Lcn972 was significantly up-regulated at 24 h, which indicated that *S. scabies* stimulated *B. velezensis* to secrete more bacteriocin.

## Discussion

Bacteriocins are considered as one of the best candidates to replace antibiotics (Tang et al., 2022), and they have been widely used in food preservation (Chandrakasan et al., 2019). In recent years, there have been many studies of bacteriocins in food and medicine (de Arauz et al., 2009; Arevalos-Sánchez et al., 2012; Gharsallaoui et al., 2013), but few applications to control plant pathogenic microbes. In our study, a 972 family lactococcin was found in *B. velezensis* HN-Q-8 and overexpressed in an *E. coli* expression system. Lcn972 was purified, and the antibacterial activity was tested. It showed a strong antibacterial activity against *S. scabies*. In addition, the stability of Lcn972 was tested, and the antibacterial mechanism of Lcn972 was initially revealed. In this study, bacteriocin was applied in the treatment of potato common scab for the first time, providing a new manner for the prevention and treatment of potato common scab.

The stability levels of bacteriocins determine whether they can fully exert theirs bacteriostatic activities and have wide applications. Lcn972 was stable at high temperatures (Figures 2D,F), and it maintained a strong activity after heating for 30 min at 121°C, which was similar to the study by Qiao et al. (2020). Generally, Class II bacteriocins have strong thermal stability (Abriouel et al., 2011), and Lcn972 is a Class II bacteriocin secreted by *B. velezensis*. Thus, it conforms to the characteristics of Class II bacteriocins. In addition, bacteriocins are highly sensitive to proteases, which can reduce the activity of bacteriocins or even completely inactivate them (Gao et al., 2010; Hammami et al., 2012; Lee and Chang, 2018; Qin et al., 2019). We determined the effects of six proteases on the activity of Lcn972 (Figure 3D). The activity of Lcn972 was significantly reduced after treatments with proteinase K, papain and alkaline protease, which was similar to previous reports. However, Lcn972 was insensitive to pepsin in our study, which may be a result of the non-acidic experimental environmental conditions (Stanforth et al., 2022).

Metal ions usually have effects on protein activity (Bousleiman et al., 2017; Kluska et al., 2018; Grazioso et al., 2020); therefore, we determined the effects of different metal ions on the activity of Lcn972 (Figure 3E). Fe^2+^ completely inhibited the antibacterial activity of Lcn972, whereas Cu^2+^ and Ca^2+^ significantly enhanced the antibacterial activity of Lcn972. Metal ions can change the conformational of a protein, which can reduce its activity, or they can act as cofactors to stabilize or even enhance protein activity (Barondeau and Getzoff, 2004; Kutyshenko et al., 2019). For example, Ansari found that Cu^2+^ causes the inactivation of bacteriocin (Ansari et al., 2018), whereas Yao found that metal ions, such as Mn^2+^, Mg^2+^, and Ca^2+^, significantly enhance protein activity (Yao et al., 2019). In our study, Cu^2+^ and Ca^2+^ may act as cofactors of Lcn972, whereas, Fe^2+^ may destroy the active center of Lcn972.

Additionally, the stability during the storage period is also an important factor in determining whether Lcn972 can be applied to the control of plant diseases. Lcn972 in our study was stable both at room temperature and 4°C (Figure 4), which may be related to our purification method. In this study, heating at 121°C was used for purification. At this temperature most proteins do not maintain their activity; therefore, there was almost no active protein remaining to degrade Lcn972.

The antibacterial mechanisms of bacteriocins differ owing to their unique structures. To date, the antibacterial mechanisms reported for bacteriocins mainly involve cell membrane perforation and the cytoplasmic leakage of the target strains, resulting in the disappearance of the proton-driven potential, thereby causing the death of the target strains (Kumariya et al., 2015; Soltani et al., 2020). Although most bacteriocins cause cell membrane perforation, their targets differ. For example, the most well-studied Class I bacteriocin nisin targets lipid II on the cell membrane (Webber et al., 2021), whereas, the targets of Class II bacteriocins are proteins related to cell membrane formation, including mannose permease, ABC transporter and undecylenyl pyrophosphate phosphatase (Suganthi and Mohanasrinivasan, 2014; Ceuppens et al., 2017; Soltani et al., 2020). In addition, Class II bacteriocins also affect RNA transcription and translation (Wang et al., 2019). In our study, Lcn972 not only damaged the cell membrane of *S. scabies* HP4, it also caused the deformation and adhesion of the hyphae, as well as damaging the cell structure of *S. scabies* HP4. This indicated that the antibacterial mechanism of Lcn972 damaged the membrane structure of *S. scabies* HP4, resulting in the destruction of the cell structure, thereby causing its death (Figures 6, 7). However, the target of Lcn972 needs to be further explored.

*Bacillus* can sense the presence of bacteria or fungi, resulting in the increased production of antibacterial substances to enhance its chance to survival (Stubbendieck and Straight, 2017). Stefanie et al. found that fungi competition enhances the production of fengycin and surfactin by *B. subtilis* (DeFilippi et al., 2018). Similarly, we found the expression of Lcn972 in *B. velezensis* HN-Q-8 was up-regulated in the presence of *S. scabies* HP4 (Figure 8B), which indicated that *B. velezensis* HN-Q-8 sensed *S. scabies* HP4 and consequently produce more Lcn972.

In conclusion, a 972 family bacteriocin Lcn972, from *B. velezensis* HN-Q-8 was studied, and it showed a strong antibacterial activity against *S. scabies* HP4 and insensitive to high temperature and UV. Additionally, Lcn972 can be stored at room temperature for a long time. Lcn972 disrupted the cell structure of *S. scabies*, resulting in cell death. Furthermore, the expression of Lcn972 in *B. velezensis* HN-Q-8 was upregulated when it sensed *S. scabies*. In the future, research will focus on searching for the target of Lcn972 and on applying Lcn972 in the control of potato common scab.

## Data availability statement

The original contributions presented in this study are included in the article/Supplementary material, further inquiries can be directed to the corresponding authors.

## Author contributions

JZu and ZY contributed to the conception of the study. JZa, XB, and BW performed the experiment. JZu, JW, and ZZ contributed significantly to analysis and manuscript preparation. JZa performed the data analyses and wrote the manuscript. DZ and LZ helped to perform the analysis with constructive discussions. All authors read and approved the final manuscript.

## Footnotes

1
https://antismash.secondarymetabolites.org/

2
https://www.uniprot.org/

3
https://www.ncbi.nlm.nih.gov/

## Supplementary material

Supplementary Figure 1 Antibacterial activity of *Bacillus velezensis* HN-Q-8 against *Streptomyces scabies* HP4.

Supplementary Figure 2 **(A)** Gene clusters of *B*. velezensis HN-Q-8. **(B)** Locus structure of Regin 6. **(C)** Domains of the RiPP-like on Regin 6. **(D)** Sequence of Lcn972 on RiPP-like.

Supplementary Figure 3 Antibacterial activity of Lcn972 on *Streptomyces scabies* HP4.

Supplementary Figure 4 Inhibitory effects of Lcn972 on bacteria.

Supplementary Figure 5 *Streptomyces scabies* treated by different concentrations of NaCl.
