# Supplementary material for: A novel of new class II bacteriocin from Bacillus velezensis HN-Q-8 and its antibacterial activity on Streptomyces scabies
Source: Front Microbiol. 2022 Jul 29;13:943232. doi: 10.3389/fmicb.2022.943232 (PMC9372549; doi:10.3389/fmicb.2022.943232)
Supplement: Supplementary file 1 [file Table_1.docx]

Table S1 Information of bacteria used in study.

| Indicator strains | Year | Source | Location | Strain number |
| --- | --- | --- | --- | --- |
| *Bacillus pumilus* G15 | 2018 | Soil | Zhangjiakou city, Hebei province, China | HAUBp0015 |
| *Bacillus subtilis* 3610 | - | - | - | ATCC 6051 |
| *Bacillus amyloliquefaciens* Z17-2 | 2017 | Soil | Zhangjiakou city, Hebei province, China | HAUBa0172 |
| *Bacillus atrophaeus* YN-29 | 2021 | Soil | Zhangjiakou city, Hebei province, China | HAUBa0029 |
| *Bacillus mycoides* Y-7 | 2016 | Soil | Baoding city, Hebei province, China | HAUBm0007 |
| *Bacillus licheniformis* J-5 | 2021 | Soil | Baoding city, Hebei province, China | HAUBl0005 |
| *Bacillus thuringiensis* 2.19 | 2020 | Soil | Qinhuangdao city, Hebei province, China | HAUBt0219 |
| *Bacillus cereus* 2.15 | 2020 | Soil | Qinhuangdao city, Hebei province, China | HAUBc0215 |
| *Bacillus velezensis* FX | 2015 | Soil | Baoding city, Hebei province, China | HAUBv0524 |
| *Bacillus mojavensis* C28 | 2018 | Soil | Zhangjiakou city, Hebei province, China | HAUBm0028 |
| *Streptomyces turgidiscabies* HY9 | 2021 | Potato | Lingyi city, Shandong province, China | HAUSt0009 |
| *Streptomyces stelliscabiei* FN1 | 2021 | Potato | Changli city, Hebei province, China | HAUSs0001 |
| *Streptomyces scabies* HP4 | 2015 | Potato | Chengde city, Hebei province, China | HAUSs0004 |
| *Streptomyces europaeiscabiei* MY | 2015 | Potato | Chengde city, Hebei province, China | HAUSe1325 |
| *Pectobacterium brasiliense* 412 | 2019 | Potato | Tangshan city, Hebei province, China | FR19412 |
| *Pectobacterium atrosepticum* B412 | 2020 | Potato | Chengde city, Hebei province, China | FN20412 |
