# Supplementary figures and images for: A novel of new class II bacteriocin from Bacillus velezensis HN-Q-8 and its antibacterial activity on Streptomyces scabies
Source: Front Microbiol. 2022 Jul 29;13:943232. doi: 10.3389/fmicb.2022.943232 (PMC9372549; doi:10.3389/fmicb.2022.943232)

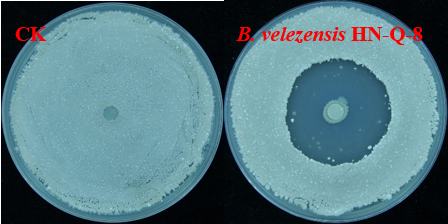

Supplement: Supplementary Figure 1 — Antibacterial activity of Bacillus velezensis HN-Q-8 against Streptomyces scabies HP4. [file Image_1.TIF]

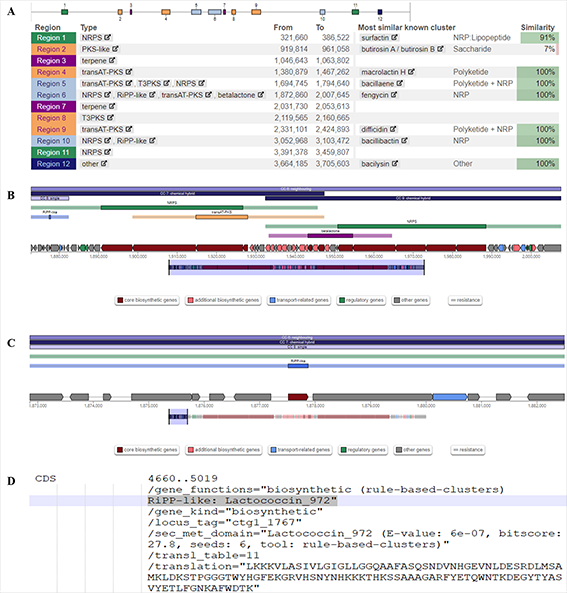

Supplement: Supplementary Figure 2 — (A) Gene clusters of B. velezensis HN-Q-8. (B) Locus structure of Regin 6. (C) Domains of the RiPP-like on Regin 6. (D) Sequence of Lcn972 on RiPP-like. [file Image_2.TIF]

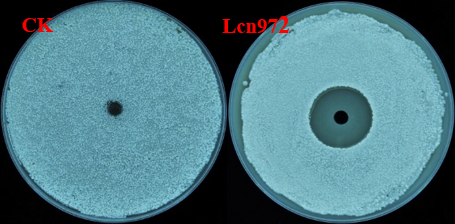

Supplement: Supplementary Figure 3 — Antibacterial activity of Lcn972 on Streptomyces scabies HP4. [file Image_3.TIF]

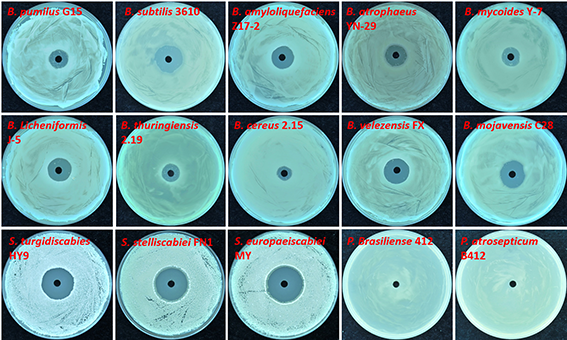

Supplement: Supplementary Figure 4 — Inhibitory effects of Lcn972 on bacteria. [file Image_4.TIF]

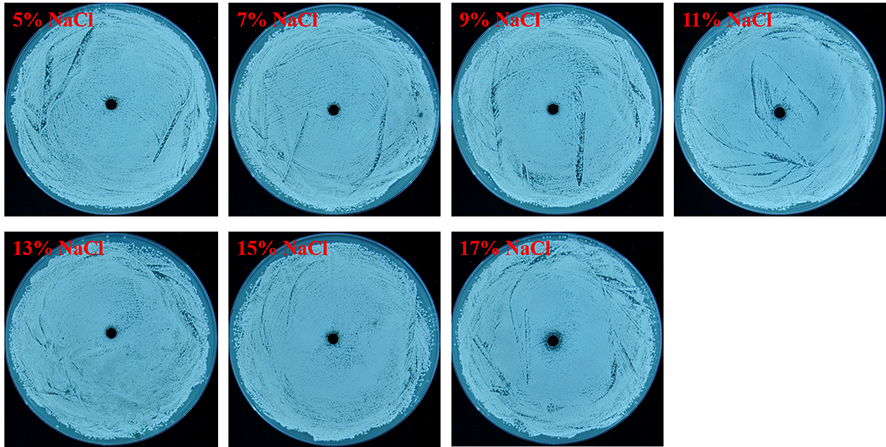

Supplement: Supplementary Figure 5 — Streptomyces scabies treated by different concentrations of NaCl. [file Image_5.TIF]
